# The Role of Protein Arginine Methyltransferases in Inflammatory Responses

**DOI:** 10.1155/2016/4028353

**Published:** 2016-03-02

**Authors:** Ji Hye Kim, Byong Chul Yoo, Woo Seok Yang, Eunji Kim, Sungyoul Hong, Jae Youl Cho

**Affiliations:** ^1^Department of Genetic Engineering, Sungkyunkwan University, Suwon 440-746, Republic of Korea; ^2^Research Institute and Hospital, National Cancer Center, Goyang 410-769, Republic of Korea

## Abstract

Protein arginine methyltransferases (PRMTs) mediate the methylation of a number of protein substrates of arginine residues and serve critical functions in many cellular responses, including cancer development, progression, and aggressiveness, T-lymphocyte activation, and hepatic gluconeogenesis. There are nine members of the PRMT family, which are divided into 4 types (types I–IV). Although most PRMTs do not require posttranslational modification (PTM) to be activated, fine-tuning modifications, such as interactions between cofactor proteins, subcellular compartmentalization, and regulation of RNA, via micro-RNAs, seem to be required. Inflammation is an essential defense reaction of the body to eliminate harmful stimuli, including damaged cells, irritants, or pathogens. However, chronic inflammation can eventually cause several types of diseases, including some cancers, atherosclerosis, rheumatoid arthritis, and periodontitis. Therefore, inflammation responses should be well modulated. In this review, we briefly discuss the role of PRMTs in the control of inflammation. More specifically, we review the roles of four PRMTs (CARM1, PRMT1, PRMT5, and PRMT6) in modulating inflammation responses, particularly in terms of modulating the transcriptional factors or cofactors related to inflammation. Based on the regulatory roles known so far, we propose that PRMTs should be considered one of the target molecule groups that modulate inflammatory responses.

## 1. Introduction

Inflammation, the body's physiological protective response to infection by pathogens, is an important component of innate immunity. Inflammation can be categorized as either acute or chronic. Recognition of pathogen-specific molecules, such as lipopolysaccharides, by pattern recognition receptors (PRRs) triggers acute inflammation; among PRRs, toll-like receptors (TLRs) have been intensively studied [1]. In response to stimulation of TLRs by an appropriate pathogen, many molecular events, including activation of nuclear factor- (NF-) *κ*B and activator protein- (AP-) 1 signal pathways, are instigated and, consequently, transcription of genes that code proinflammatory enzymes, such as inducible nitric oxide (NO) synthase (iNOS) and cyclooxygenase- (COX-) 2, is increased [2, 3]. iNOS-induced NO and COX-2-derived prostaglandin E_2_ (PGE_2_) act as key mediators of active inflammation, affecting essentially all stages of the inflammation process [4, 5]. Because acute inflammation, a generic response that attempts to remove the initial cause of infection, requires constant stimulation to be maintained, it begins to attenuate as stimuli decline [6, 7]. A serious complication comes up during long-lasting inflammation condition, known as chronic inflammation. Chronic inflammation can cause inflammatory-related or autoimmune diseases, including rheumatoid arthritis, Alzheimer's disease, systemic lupus erythematosus, asthma, atherosclerosis, cancer, and ischemic heart disease [8, 9].

Biologically, methylation is a reaction that adds a methyl group to substrates, including DNA, RNA, and proteins, via various methyltransferases. DNA methylation mainly occurs at cytosine-phosphate-guanine (CpG) sites, where a cytosine follows a guanine in the DNA sequence. The cytosine in CpG sites is methylated by DNA methyltransferases to form 5-methylcytosine. In mammals, 70 to 80% of CpG cytosines exist as methylated form, and CpG methylation is a key reaction in epigenetics because it acts as a switch that turns a gene on or off. Additionally, protein methylation, a posttranslational modification, is a reaction that covalently adds a methyl group to specific amino acid residues; these reactions can be divided into two main categories:* N*-methylation and* O*-methylation (carboxymethylation). The methylation of lysine, arginine, histidine, alanine, proline, glutamine, phenylalanine, asparagine, and methionine is a type of* N*-methylation, while* O*-methylation involves the methylation of glutamic acid and aspartic acid. The creation of these methylated amino acids is catalyzed by methyltransferases that utilize S-adenosyl-L-methionine (AdoMet) as the methyl donor. Three types of AdoMet-dependent methyltransferases are defined, based on their structural features. The largest class (class I) has a seven-strand twisted *β*-sheet structure [10]. Class II encompasses SET lysine methyltransferases [11]. Finally, class III contains membrane-associated enzymes [12].

**Figure 1:**
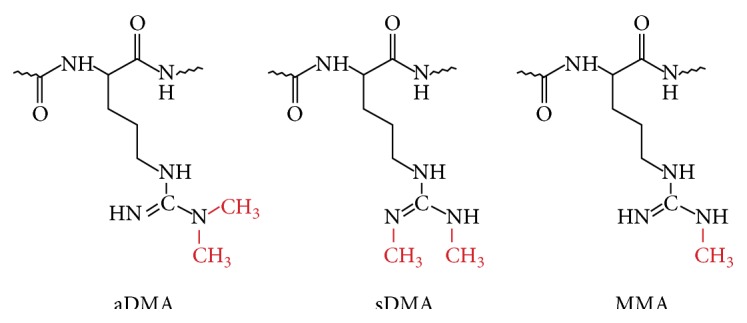
Conformation of three different methylarginines.

Arginine methylation is catalyzed by the protein arginine-N-methyltransferase (PRMT) family and is observed in both cytoplasmic and nuclear proteins. Methylation of arginine residues is involved in many cellular responses, including RNA splicing, signaling transduction, DNA damage repair, and protein-protein interactions. The functions of arginine-methylated proteins are listed in Table 1. Arginine is methylated in three different ways: monomethylated arginine (MMA), symmetrically dimethylated arginine (sDMA), and asymmetrically dimethylated arginine (aDMA). MMA is considered an intermediate form of DMA. Each type of methylarginine is produced by one of nine PRMTs (Figure 1).

**Figure 2:**
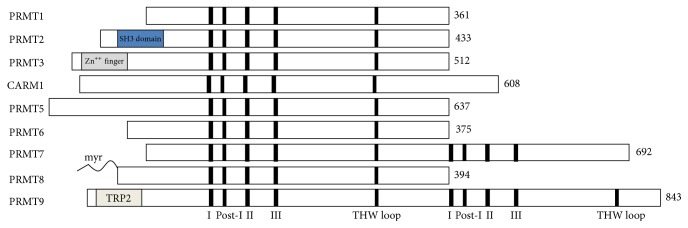
Structure of the PRMT family.

PRMT family members are a class I enzyme, having a set of four signature motifs (I, post-I, II, and III) and a conserved THW loop [13]. Motif I, post-I, and the THW loop are important to the formation of the AdoMet-binding pocket (Figure 2) [14]. Glycine- and arginine-rich patches (GAR motifs) in substrates are mainly methylated by PRMTs, but coactivator-associated arginine methyltransferase 1 (CARM1) is an exception. PRMTs are divided into 4 types (types I–IV) of enzymes (Figure 2). Type I arginine methyltransferase, the most common type of PRMT, induces asymmetric dimethylation, adding two methyl groups to the terminal nitrogen atoms (*ω*-N^G^,N^G^-dimethylarginine). Six enzymes are categorized as type I PRMTs: PRMT1, PRMT2, PRMT3, PRMT4, PRMT6, and PRMT8 [15]. Among them, PRMT1 is the predominant type I enzyme. Type II PRMTs add one methyl group to the terminal nitrogen atoms (*ω*-N^G^,N^G^-dimethylarginine) by catalyzing the symmetric dimethylation of arginine side chains. PRMT5 is associated with this type, whereas PRMT7 is thought to belong to type II, although this is still controversial [16]. Recently, PRMT9 turned out to belong to the type II enzyme that methylates RNA splicing factor SF3B2 [17, 18]. Type III enzymes generate monomethyl arginine as their final product, even though monomethylated arginine at terminal nitrogen atoms (*ω*-N^G^-methylarginine) is an essential intermediate of both types I and II PRMT reactions [19]. Type IV methyltransferases catalyze monomethylation of the internal nitrogen atom (N-methylarginine), which is found only in fungi (Table 2) [20].

Most PRMTs are active when they are expressed as purified recombinant proteins, which indicates that PRMTs do not require additional processing or PTMs to maintain their activity. However, there are mechanisms for fine-tuning PRMT activity, such as PTMs, interacting with regulatory proteins, subcellular compartmentalization, and control of RNA levels by micro-RNAs. Regulation of PRMTs is explained in Table 3.

## 2. The Role of PRMTs in Inflammatory Responses

According to a number of recent reports, four PRMTs are currently thought to be correlated with inflammatory responses: CARM1, PRMT1, PRMT5, and PRMT6.

### 2.1. CARM1 (PRMT4)

CARM1, also known as PRMT4 (protein arginine N-methyltransferase 4), regulates many proteins involved in DNA packing, transcription regulation, pre-mRNA splicing, and mRNA stability by inducing methylation of the guanidine nitrogen of arginine residues of substrates. There are two classes of CARM1 substrates: chromatin remodeling proteins (histone H3 and p300/CBP), which are included in class 1 substrates, and proteins possessing RNA-binding activity, such as PABP1, TARPP, HuR, HuD, and splicing factors [46]. CARM1 acts as a secondary coactivator and is associated with the p160 family (SRC-1, GRIP1, and AIB) of transcriptional coactivators, which are involved in gene activation by steroid hormone receptors [47]. CARM1 also associates with CBP/p300 transcriptional coactivators that activate steroid hormone receptors and C/EBP-mediated gene expression; it functions as a coactivator or corepressor of CBP/P300 molecules. As a coactivator, CARM1 is recruited to nuclear receptors by p160 coactivator, which is activated by hormone. Chromatin remodeling occurs through histone acetylation and methylation proximate to the hormone response element (HRE); as a consequence, transcription is stimulated. In contrast, CARM1 acts as a corepressor of cyclic AMP-induced signaling when accompanied by CBP/p300. CARM1-methylated CBP was found to inhibit transcriptional activity of CREB by blocking the interaction between the KIX domain (the CREB and MYB interaction domain in CBP) and the kinase-inducible domain (KID) of CREB [48].

#### 2.1.1. The Role of CARM1 in Inflammation through the Regulation of NF-*κ*B

Nuclear factor- (NF-) *κ*B is one of the most important transcriptional factors because it regulates the transcription of many proteins involved in inflammatory diseases, autoimmune diseases, septic shock, viral infection, and immune development. Indeed, elevated NF-*κ*B activity has been detected in the airways of asthmatic patients [49]. Increased expression of NF-*κ*B has been noted in Crohn's disease and ulcerative colitis patients [50]. Additionally, upregulated NF-*κ*B was observed in the synovial fluid of patients with rheumatoid arthritis [51]. The transcription factor, NF-*κ*B, is composed of homo- or heterodimers of subunits (members of the Rel family), including p50, p52 (p49), p65 (Rel A), c-Rel, and Rel B. All these proteins contain a highly conserved domain known as the Rel homology domain (RHD), which consists of about 300 amino acids and plays a role in homologous or heterologous dimerization, DNA binding, and nuclear translocation. Among the various NF-*κ*B subunit combinations, the p65/p50 and c-Rel/p50 heterodimers are the most commonly described forms.

**Figure 3:**
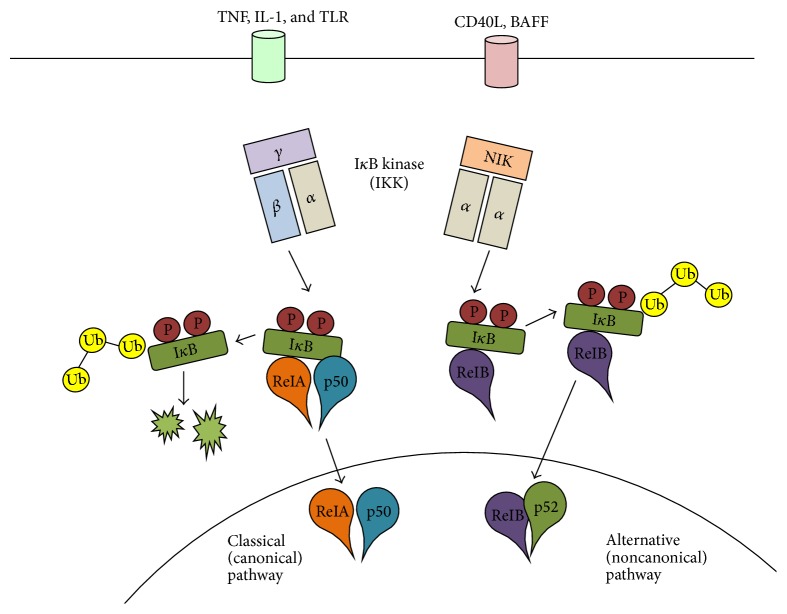
Two pathways leading to NF-*κ*B activation.

There are two pathways leading to NF-*κ*B activation: the classic (canonical) pathway and the alternative (noncanonical) pathway (Figure 3) [52]. The pathways are defined based on different requests for IKK subunits that regulate NF-*κ*B activation at an upstream stage. The IKK complexes are composed of three subunits, including IKK*α* (IKK1) and IKK*β* (IKK2), which are kinase subunits, and a regulatory subunit IKK*γ* (NEMO). In the canonical pathway, IKK*β* and IKK*γ*, but not IKK*α*, regulate degradation of I*κ*B through phosphorylation of I*κ*B and, consequently, free NF-*κ*B is translocated into the nucleus [53]. The alternative pathway, however, requires only IKK*α*, in which p100, a precursor of p52, is phosphorylated and matured by IKK*α* [54]. The stimuli of each pathway are also different. The major triggers for the canonical pathway are proinflammatory cytokines and microbial products, such as tumor necrosis factor- (TNF-) *α*, IL-1, and lipopolysaccharide (LPS), resulting in activation of complexes comprised of Rel A or c-Rel [55], whereas the alternative pathway is activated by the TNF family, leading to activation of Rel B/p52 complexes [56].

There are coactivators of NF-*κ*B, CREB-binding protein (CBP), and its homolog, p300, including the p300/CBP-associated factor (P/CAF) and members of the steroid receptor coactivator (p160/SRC) family. These coactivators directly interact with NF-*κ*B subunits, such as p50 and p60 [57]. Interestingly, CARM1 is closely associated with the transcriptional coactivator, CBP/p300, in p53-mediated and nuclear receptor- (NR-) mediated transcriptional regulation. This implies that CARM1 would be a coactivator of NF-*κ*B, because NF-*κ*B utilizes a similar set of coactivator proteins with p53 and NR [58]. Indeed, CARM1 was found to be a novel transcriptional coactivator of NF-*κ*B [59]. The NF-*κ*B-dependent genes, such as ICAM-1, G-CSF, MCP-1, IP-10, and MIP-2, were impaired in Carm1(−/−) fibroblasts that underwent TNF-*α* and LPS stimulation. TNF-*α*- and LPS-induced NF-*κ*B reporter gene activities were also lower in Carm1(−/−) cells compared to controls. However, I*κ*B degradation and p65/Rel A translocation were not affected by the absence of CARM1. Instead, it seems that CARM1 regulates NF-*κ*B-mediated gene expression through complex formations with p65 and p300. Consequently, CARM1 acts as a primary coactivator by enhancing NF-*κ*B recruitment to cognate sites and by controlling transcription in a gene-specific manner [59].

However, CARM1's functional role in NF-*κ*B-dependent gene expression remains unclear and even controversial. According to the recovery experiments using complemented* carm1−/−* mouse embryonic fibroblast cells by retroviral transduction, either with wild-type CARM1 or with an enzymatic inactive E267Q mutant of CARM1, CARM1 enzymatic activity was not essential for NF-*κ*B-dependent gene expression which was stimulated by TNF-*α* or PMA. Additionally, CARM1 is not needed for recruitment of Rel A/p65 to chromatin, indicating that CARM1 contributes to the stabilization of complex proteins. These observations generate two hypotheses: (1) CARM1 might recruit Brg1, an enzymatic ATPase subunit of the SWI/SNF complex, to promoters of specific genes, because CARM1 interacts with Brg1 [60]; (2) a third interaction partner, whose enzymatic activity is independent of CARM1, might also be recruited by CARM1. Therefore, a more rigorous investigation of CARM1's role in transcriptional regulation is required to understand its exact role in inflammatory responses.

### 2.2. PRMT1

PRMT1 is the most common form of PRMT and is expressed in most tissues, constituting up to 85% of all PRMT activity in cultured RAT1 cells and in mouse liver tissue under experimental conditions [61]. PRMT1 is broadly thought to be the main enzyme on histone H4 for monomethylation and asymmetric dimethylation of Arg-3, which are required for transcriptional activation by nuclear hormone receptors [62]. Nonhistone proteins have also been reported to act as substrates of PRMT1. Through the methylation of PIAS1, PRMT1 can control STAT1 transcriptional activity in the late phase of interferon-*γ* (IFN-*γ*) signaling [63]. PRMT1 acts as an activator of estrogen receptor- (ER-) mediated transactivation through TAF15 methylation [64]. Also, PRMT1 methylates FOXO1 and increases its transcriptional activity by retaining it in the nucleus [65].

#### 2.2.1. The Role of PRMT1 in Antigen-Induced Pulmonary Inflammation (AIPI) in Rats

An antigen-induced pulmonary inflammation (AIPI) model is the* in vivo* rat asthma model that shares many pathological features with human asthma. Interestingly, there are remarkable differences in the gene expression of PRMTs in rats with AIPI comparing to normal rats [66]. In particular, the expression of PRMT1 was significantly higher in the AIPI model, implying putative involvement of PRMT1 in asthma.

During pulmonary inflammation, eosinophils, the most critical immune cells in asthmatic conditions, are recruited into the lungs through a process mediated by eotaxins. Interleukin- (IL-) 4 boosts eosinophilic inflammation by producing eotaxin-1 [67]. PRMT1 has been shown to be associated with the mechanisms underlying the recruitment of eosinophils into airways by IL-4 [68]. The upregulation of PRMT1 was induced by Th2 cytokine IL-4 in the AIPI model. According to a transcription factor search program, IL-4 seems to increase PRMT1 expression through activation of STATs, CREB, NF-*κ*B, and GATA3, which are all involved in the promoter region of PRMT1. AMI, a PRMT1 inhibitor, suppressed eotaxin-1 production and eosinophil infiltration in the AIPI model, implying that PRMT1, when activated by IL-4, functions as a pulmonary inflammation regulator via the modulation of eotaxin-1 release [68]. It has also been reported that PRMT1 can methylate the STAT family, which is responsible for IL-4 expression [69]. Therefore, it has been suggested that IL-4 induces overexpression of PRMT1, leading to increased transcription of eotaxin-1 by elevated STAT signaling. Additionally, transforming growth factor- (TGF-) *β*-induced PRMT1 also contributes to pulmonary inflammation in chronic AIPI. TGF-*β* is produced by IL-4 stimulated epithelial cell, and subsequently proliferation of fibroblast and PRMT1 expression are elevated. Then, increased PRMT1 is regarded to regulate pulmonary inflammation through inducing COX-2 expression [70].

#### 2.2.2. Regulation of CITED2 by PRMT1

CBP/p300-interacting transactivator 2 (CITED2) is a coactivator of the p300/CBP-mediated transcript complex. It also acts as a transcriptional corepressor of HIF-dependent genes [71]. It serves multiple functions in several biological processes; for example, in knockout analyses, CITED2 was observed to play important roles in mouse embryo development [72–74]. Moreover, CITED2 is required for maintenance of adult hemopoietic stem cell functions [75]. A recent study on CITED2 function in immunity and inflammation led to the observation that CITED2 is induced by LPS and acts as a novel repressor of NF-*κ*B by preventing p65 from binding to p300 [76].

Interestingly, PRTM1 and CARM1 regulate CITED2 expression under IL-4 stimulation conditions. According to Uta-Maria's work, PRMT1 and CARM1 were observed to recruit CITED2 gene promoter sites when stimulated with fetal calf serum (FCS)/IL-4 [77]. Additionally, both PRMTs interact with STAT5 in an IL-4-dependent manner [77]. The data indicate that PRMT1 and CARM1 cooperatively increase the expression of CITED2 through STAT5-dependent transcriptional activation when induced by IL-4 signaling. Interaction of the two PRMTs with STAT5 and their recruitment to the promoter region are also enhanced by IL-4 stimulation [77]. These findings imply that CARM1 and PRMT1 might participate in immune responses by regulating CITED2 transcription.

#### 2.2.3. Modulation of NF-*κ*B by PRMT1

PRMT1 is considered an inflammation regulator because of its NF-*κ*B regulation capacity. PRMT1 controls NF-*κ*B-dependent gene expression in collaboration with other coactivators. Hassa et al. found that, under TNF-*α* stimulation, PRMT1 forms a nuclear complex with p65 and poly[ADP-ribose] polymerase 1 (PARP1), and PRTM1 is recruited to p65-containing complexes that are associated with promoters [78]. Moreover, PRMT1 was required for PARP1 and p300-dependent NF-*κ*B gene transcription, based on luciferase reporter gene assays, suggesting that PRMT1 synergistically coactivates NF-*κ*B-dependent transcription in cooperation with PARP1 and p300.

How these coactivators (PRMT1, PARP1, and p300) cross-talk as part of their associated activity is still unclear. One possible explanation is that histone acetylation by CBP/p300 might be succeeded by PRMT1-mediated methylation of Arg-3 on histone H4, because it has been revealed that Arg-3 methylation on H4 by PRMT1 is essential to maintain “active” chromatin modification [79]. Another possibility is that PRMT1 may methylate other NF-*κ*B transcriptional coactivators; for example, CARM1 catalyzes p300/CBP methylation, which is linked to the alteration of transcription states [48]. Similar to CARM1, PRMT1 might directly methylate promoter-associated coactivators, such as PARP1 or p160 family members. This explanation is consistent with Stallcup et al.'s findings regarding the functional regulation of nonhistone proteins by PRMT1 [80]. Further studies are needed to fully understand how PRMT1 regulates NF-*κ*B-dependent gene expression through its collaboration with other cofactors.

### 2.3. PRMT6

Protein arginine methyltransferase 6 (PRMT6) mainly catalyzes asymmetric dimethylation of histone H3 Arg-2 (H3R2me2a). Histone methylation by this enzyme has been confirmed* in vivo* [81]. Thus, alteration of PRMT6 expression could affect universal gene expression. In addition to methylation on histone proteins, PRMT6 has been observed to control gene expression by direct interaction with transcription factors, including NF-*κ*B and G-protein pathway suppressor 2 (GPS2). Because those two molecules are directly involved in inflammatory responses, it is possible that PRMT6 also plays a role in inflammation responses.

#### 2.3.1. Regulation of NF-*κ*B via PRMT6

In total, 4 PRMTs (PRMT1, CARM1, PRMT5, and PRMT6) are known to be regulators of NF-*κ*B, despite having different regulatory mechanisms. The role of PRMT6 in NF-*κ*B was first described by Di Lorenzo et al., using a gain-of-function mouse model [82]. This group established a transgenic mouse model that overexpressed PRMT6 and explored the role of PRMT6 in inflammatory responses in these mice. They observed that PRMT6 elevated IL-6 expression levels by regulating NF-*κ*B. Detailed experiments led to the conclusion that PRMT6 directly binds to the NF-*κ*B subunit, Rel A, allowing shuttling of Rel A into the nucleus. Consistent with this, colocalization of Rel A and PRMT6 at NF-*κ*B binding promoters was observed to be elevated and, subsequently, NF-*κ*B target gene expression, including IL-6, was elevated when stimulated by TNF-*α*.

However, the role of arginine methylation by PRMT6 during activation of NF-*κ*B-related gene expression has not been thoroughly explored. According to* in vitro* methylation assay results, PRMT6-mediated Rel A methylation was not directly observed, indicating that NF-*κ*B activation is indirectly regulated by methylation of NF-*κ*B coactivators, such as p160/steroid receptor coactivator (SRC) proteins. Indeed, PRMT6 interacts with activation domain 2 (AD2) of SRC-1, increasing the possibility that p160/SRC could be methylated by PRMT6 [83].

#### 2.3.2. Regulation of G-Protein Pathway Suppressor 2 (GPS2) by PRMT6

GPS2 is a multifunctional protein belonging to a transcriptional cofactor. GPS2 serves a function in G-protein mitogen-activated protein kinase (MAPK) signaling pathways, appearing to have a negative effect on RAS-, MAPK-, and JAK-mediated signaling cascades. Because these enzymes are major signaling regulators associated with inflammatory responses, the modulation of GPS2 by PRMT6 implies involvement of PRMT6 in controlling inflammation [84].

Recently, Huang et al. observed that PRMT6 modulates GPS2 by arginine methylation at Arg-323 and Arg-312 [85]. Of these, Arg-323 methylation was found to be an essential reaction that prevented proteasomal degradation of GPS2, resulting in its increased stability. Huang et al.'s findings indicate that methylation of Arg-323 is needed for recognition by transducer beta-like protein 1 (TBL1), which prevents degradation of polyubiquitinated GPS2. TBL1 subsequently binds to the ubiquitinated GPS2 by recognizing the methylated Arg-323, which was catalyzed by PRMT6. Consequently, proteasomal degradation is diminished. Although the direct relationship between inflammation and PRMT6 and GPS2 is still unclear, accumulated reports indicate a strong likelihood that PRMT6 is an inflammation regulator.

### 2.4. PRMT5

Protein arginine methyltransferase 5 (PRMT5) catalyzes the production of a symmetrically dimethylated (SDMA) guanidine group and belongs to a type II PRMT. Similar to other PRMTs, PRMT5 also controls gene expression, mainly by modulating histone methylation. PRMT5 represses gene transcription by inducing dimethylation of Arg-8 on histone H3 (H3R8) and Arg-3 on histone H4 (H4R3) [86]. Contrary to this, PRMT5 can also upregulate gene transcription under particular conditions [87].

#### 2.4.1. The Role of PRMT5 in HOXA9-Mediated Endothelial Cell (EC) Inflammation

EC inflammatory responses are mediated by proinflammatory endothelial-leukocyte adhesion molecules (ELAM), such as E-selectin and vascular cell adhesion molecule 1 (VCAM-1) [88]. When ECs receive inflammation signals, transcription factors that induce adhesion molecules are activated [89]; a key transcriptional factor involved in this reaction is HOXA9 [90]. HOXA9 belongs to the homeobox family, and its posttranslational modifications, including phosphorylation and ubiquitination, play a critical role in hematopoietic differentiation [91]. Interestingly, HOXA9 is methylated at Arg-140 by PRMT5 and this reaction is activated by TNF-*α*. Methylation of HOXA9 plays a critical role in the upregulation of ELAM (E-selectin and VCAM-1), indicating that PRMT5 could play an important role in EC inflammation [92]. In contrast, PRMT5 has an inhibitory role in the induction of E-selectin by mediating histone H4R3, which leads to gene silencing [93]. Therefore, it seems that PRMT5 could contribute to EC inflammation as an on-off switch.

#### 2.4.2. The Function of PRMT5 in Immune Responses through NF-*κ*B Regulation

There are several reports indicating that PRMT5 regulates NF-*κ*B activity. At first, PRMT5 was reported to be a NF-*κ*B regulator during TRAIL-induced apoptosis [94]. According to Hiroshi et al., PRMT5 binds to the TRAIL receptor and, consequently, TRAIL-induced apoptosis is activated via IKK activation and I*κ*B degradation. Because TRAIL may stimulate inflammatory cytokine expression, such as CCL20, and because NF-*κ*B is a key regulator of inflammation in immune cells, PRMT5 involvement in inflammatory responses was also explored. In practice, PRMT5 appears to be associated with DR4-dependent immune regulation by controlling the NF-*κ*B pathway [95]. DR4 binds to TRAIL, leading to recruitment of RIP1 and TRAF2 in DISC, as well as activation of NF-*κ*B. In these reactions, PRMT5 acts as a competitor of TRAIL to bind to DR4, resulting in suppression of NF-*κ*B activation and CCL20 expression.

Additionally, PRMT5 was directly revealed to regulate NF-*κ*B activity by inducing methylation of the p65 subunit. Wei et al. demonstrated that PRMT5 methylates Arg-30 (R30) residues of the p65 subunit and regulates NF-*κ*B-dependent gene expression, such as interleukin-1*α* (IL-1*α*) and TNF receptor-associated factor 1 (TRAF1). According to microarray analyses, about 85% of NF-*κ*B-dependent gene expression seems to be required for R30 and p65 methylation [96]. With that, it has also been reported that R35, as well as R30, is methylated by PRMT5. Methylated R30 and R35 at p65 participate in elevating p65 and in transcription of a subset of TNF-*α*-induced proinflammatory genes, especially CXCL10, in endothelial cells [97].

## 3. Conclusions

Based on our review, there is evidence for a correlation between PRMTs and inflammatory responses. In particular, transcription regulation by NF-*κ*B, a key molecule of inflammation, appears to be a main function of PRMTs in the regulation of inflammation system. However, studies linking PRMTs to inflammation are in a very nascent stage, and the current evidence is circumstantial. Therefore, an introduction of key model systems is needed in order to understand the biological role of PRMTs in inflammation. Mouse knockout models could be useful in this process (Table 4). In fact, mouse models can be a powerful tool for investigating the* in vivo* inflammation-regulatory roles of PRMTs and understanding their molecular mechanisms. The relevance of the association between PRMTs and inflammatory diseases can also be estimated using* in vivo* inflammatory knockout models, such as hepatitis, gastritis, colitis, peritonitis, and dermatitis. Additionally, phenomenological access is likely to be required to understand the connection between PRMTs and inflammatory disease. For that, a good approach would be to examine the expression pattern of PRMT activity in immune cells obtained from* in vivo* inflammatory disease models or chronic inflammatory disease patients. Moreover, testing the inhibitory efficacy of PRMT inhibitors against inflammatory diseases will contribute to the development of a new anti-inflammatory drug.

Lastly, the possibility of functional involvement of arginine demethylase in arginine methylation cannot be excluded. Many studies have indicated the existence of arginine demethylases, even though arginine methylation is a stable modification [98]. For example, H3R17me2A exhibits cyclic expression with 20-minute fluctuation intervals [99], and PRMT1-induced transient methylation of ER*α* with estrogen treatment reaches peak levels within 5 minutes and then disappears within 10 minutes [100]. In practice, Jumonji C domain-containing protein 6 (JMJD6), also known as a lysine hydroxylase, was identified as a first putative arginine demethylase [101]. Therefore, recent reports on PRMTs and their counterpart arginine demethylase propose that these enzymes are a functionally important unit in the regulation of inflammatory responses. Further verification of involvement of these enzymes in each inflammatory disease will be of considerable interest.

## Figures and Tables

**Table 1:** The biological function of methylated arginine.

Postulated or proven function	Proteins involved
mRNA splicing	Motor neuron proteins Small nuclear ribonucleoprotein
Signal transduction	Interferon receptor
Cellular proliferation	Transcription factor
Chromatin remodeling	Histones
Transcriptional coactivator	Nuclear receptor, p53
Protein-protein interaction	Inter- and intramolecules
Translocation	hnRNP
Myelogenesis	Myelin basic protein

**Table 2:** Classification of the PRMT family.

PRMTs	Family	Methylarginine formation by PRMTs
PRMT1	Type I	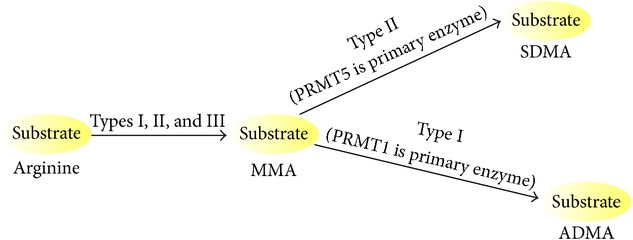
PRMT2	Type I	
PRMT3	Type I	
PRMT4 (CARM1)	Type I	
PRMT5	Type II	
PRMT6	Type I	
PRMT7	Type II (controversial) and type III	
PRMT8	Type I	
PRMT9	Type II	

**Table:** (a) PTMs: PRMT activities are modulated by PTMs

PRMTs	Types of PTM	Enzymes involved in PTM	Biological role of PTM	Reference
CARM1	Phosphorylation at S217	Unidentified kinase	Activating by regulation to bind with AdoMet	[21, 22]
	Glycosylation	N-acetylglucosamine transferase (OGT)	Activating by controlling the phosphorylation of CARM1	[23, 24]
	Auto-arginine methylation	Unidentified	Unclear	[25]

PRMT5	Amino-terminal phosphorylation	Janus kinase 2 (JAK2)	Inactivating via blocking the interaction between PRMT5 and methylosome protein 50 (MEP50)	[26]

PRMT6 and PRMT8	Auto-arginine methylation	Unidentified	Unclear	[27, 28]

**Table:** (b) Regulatory partner: interaction with regulatory proteins can control the activity of PRMTs

PRMTs	Regulatory partner	Biological role	Reference
PRMT5	MEP50	Required for PRMT5 activation	[29]
	SWI/SNF chromatin complex	Elevates the activity of MEP50-PRMT5 toward histone substrates	[30]
	Exon junction complex component and RNA-binding protein Y14	Enhances MEP50-PRMT5 activity toward Sm proteins	[31]

PRMT1	Orphan nuclear receptor TR3 (NR4A1)	Inhibits PRMT1 enzyme activity	[32]
	BTG1-binding chromatin assembly factor 1 (CAF1)	Negatively regulates PRMT1 activity	[33]
	BTG1	Increases PRMT1 activity	[34]
	PRMT2	Stimulates PRMT1 activation	[35]

PRMT3	Tumor suppressor DAL1	Blocks PRMT3 methyltransferase ability	[36]

PRMT7	CCCTC-binding factor like (CTCFL)	Increases PRMT7 activity	[37]

**Table:** (c) miRNA regulation

PRMTs	Type of miRNA	Reference
PRMT5	miR-19a, miR-25, miR-32, miR-92, miR-92b, and miR-96	[38]

**Table 4:** PRMTs of knockout mice.

PRMT	Knockout mouse phenotype	Reference
*Prmt1*	Embryonic lethality	[39]
	Loss of PRMT1 in mouse embryonic fibroblasts (MEFs) leads to spontaneous DNA damage, cell cycle progression delay, checkpoint defects, aneuploidy, and polyploidy	[40]

*Carm1*	Neonatal lethality	[41]
	Mutant embryos have defects in many systems, including adipose tissue, hematopoietic system, immune system (T-cell differentiation), and the respiratory system	[42]

*Prmt5*	Early embryonic lethality (dies by E6.5)	[43]
	PRMT5 is required for NPC homeostasis	[44]

*Prmt6*	No lethality	[45]
	MEFs from PRMT6(−/−) mice show growth defects and undergo cellular senescence	
